# Effects of Dual Exposure to the Herbicides Atrazine and Paraquat on Adult Climbing Ability and Longevity in *Drosophila melanogaster*

**DOI:** 10.3390/insects10110398

**Published:** 2019-11-10

**Authors:** Pamela C. Lovejoy, Anthony C. Fiumera

**Affiliations:** Department of Biological Sciences, Binghamton University, Binghamton, NY 13902, USA; pam.c.lovejoy@gmail.com

**Keywords:** Antagonism, Herbicides, Mixtures, Pesticides, Stressors, Synergy

## Abstract

Anthropomorphic effects are changing the planet, and therefore, organisms are being exposed to many new biotic and abiotic stressors. Exposure to multiple stressors can affect organisms in ways that are different than the sum of their individual effects, and these interactions are often difficult to predict. Atrazine and paraquat are two of the most widely used herbicides in the United States, and are individually known to increase oxidative damage, affect dopaminergic functioning, reduce longevity, and alter motor ability in non-target organisms. We measured the effects of individual and combined exposure to low doses of atrazine and paraquat on climbing ability and longevity of *Drosophila melanogaster*. Atrazine and paraquat interact to affect *D. melanogaster* climbing ability and longevity in different ways. Atrazine appeared to have a weak mitigative effect against the decrease in climbing ability caused by paraquat. In contrast, combined exposure to atrazine and paraquat had detrimental synergistic effects on female longevity. Overall, this study shows that atrazine and paraquat can interact and that it is important to measure several traits when assessing the consequences of exposure to multiple stressors. Future studies should continue to assess the impacts of stressor interactions on organisms, as many combinations have never been examined.

## 1. Introduction

Global changes due to human activity are occurring [1]. To better foresee their effects, we need a more thorough understanding of how species interact with their environments and respond to existing and developing environmental stressors, such as rising temperatures, biological invasions, habitat destruction, and chemical pollution [2,3]. Although it is critical to study the effects of exposure to individual stressors, investigating the potential for interactions between different stressors is equally as important. 

Interactions between stressors are often unexpectedly complex [2,3,4,5]. When the combined effect of stressors is equal to the sum of their individual effects, the stressors are said to act in an additive manner. If the combined effect is somehow different than the sum of their individual effects, the stressors are said to act in an interactive manner. Just as the presence of an interaction can be unpredictable, how those interactions manifest can also be complex. Synergism occurs when the combined effect of multiple stressors is greater than the sum of the individual effects, while antagonism occurs when the combined effect is less than the sum of the individual effects [6]. Synergistic and antagonistic interactions have been observed in nature between biotic and/or abiotic stressors [2,4,6]. 

Atrazine and paraquat, two commonly applied herbicides in the United States, are used in overlapping regions [7], such that organisms in these areas can be exposed to both toxicants. While, the use of atrazine per year has remained relatively constant at about 70 million pounds, the use of paraquat has increased dramatically in recent years, from over four million pounds in 2011 to about nine million pounds in 2016 [7], thereby increasing the chances of exposure to both. Atrazine, a triazine herbicide, has negative effects on various traits in both vertebrates [8,9,10,11] and invertebrates [12,13,14]. In the fruit fly, *Drosophila melanogaster,* atrazine causes differential expression of proteins associated with oxidative stress [15], increases oxidative damage and reactive oxygen species generation, alters antioxidant gene expression [13], reduces longevity [14], and reduces male reproductive fitness [16]. Paraquat, a bipyridinium herbicide, is a well-known inducer of mitochondrial oxidative stress [17]. In *D. melanogaster*, paraquat exposure reduces longevity [18,19,20], decreases dopaminergic neuron number [18,21], changes dopamine and dopamine metabolite levels [21], and alters motor functions [18,19,22]. Both atrazine and paraquat are known to interact with other environmental stressors [23,24,25,26,27]. To our knowledge, however, no other study has specifically examined the combined effects of atrazine and paraquat in vivo. Since organisms are frequently exposed to multiple stressors [2,4], it is critical to understand how these stressors might interact to affect a number of ecologically important traits.

The goal of this study is to investigate the effects of dual exposure to two commonly used herbicides, atrazine and paraquat, on climbing ability and longevity of *D. melanogaster.* This system can serve as a model to help us better understand the consequences exposure to multiple stressors and how anthropomorphic changes can affect traits related to fitness.

## 2. Materials and Methods

### 2.1. Drosophila Lines and Husbandry

All flies used in this experiment were Oregon-R (ORE-R), an inbred but robust, wild type line. The flies were maintained on a 12 hour light/dark cycle in an incubator, at 25 °C, on a standard yeast/dextrose media [28]. Adult flies, with no history of exposure to either atrazine or paraquat, laid eggs on either control food or food containing 2 ppM (9.27 µM) atrazine (Sigma Aldrich, St. Louis, MO, USA) [16] for one to two days and were then discarded. Virgins were collected within eight hours of eclosion and housed on the same food type, in single sex groups of 10 individuals, for four to five days. The flies were starved in empty vials for four hours and then exposed to paraquat (Sigma Aldrich, St. Louis, MO, USA) on gel blot paper (Whatman GB 003, Sigma Aldrich; 0.5 mL on ~1.2 cm × 5 cm strips) at either 200 µM (5.14 ppM) or 5 mM (128.58 ppM) in 1% sucrose or a 1% sucrose control for 24 hours. In total, there were six possible treatment groups (Table 1).

In this study, we were interested in the effects of low-dose herbicide exposure occurring as agricultural concentrations dilute. In an agricultural setting, the concentrations can be much higher, with maximum manufacturer suggested active ingredient concentrations of 23,965.3 ppM for atrazine and 1997.5 ppM for paraquat. The concentrations used may be lower than this, depending on the volume of liquid used for application. A concentration of 2 ppM atrazine was chosen for this study because it consistently has effects in flies in our lab at this concentration when compared to a range of doses between 2 ppB and 20 ppM [14,16,29]. Concentrations under 3 ppB are considered safe by the US EPA and 20 ppM is the maximum concentration that atrazine will dissolve in water. Concentrations of 200 µM and 5 mM paraquat were chosen because they showed respectively mild and strong effects on flies after 24 hours, compared to similar concentrations [18], as well as based on our own observations of the effects on climbing ability when exposed during adulthood [30]. Atrazine was administered in the food and paraquat was administered via gel blot paper because of the precedent set by our lab [14,16,29,30]. Paraquat was not incorporated into food because of the inhalation risk to experimenters. While, atrazine could have been fed to the flies on gel blot paper as with paraquat, we did not think such a short exposure would have much of an effect on the flies, based on its minimal effect on climbing ability after an extended exposure, as seen in the present study. It is possible, however, that a short adult exposure to both herbicides at once could have the same effects that we observed in this study. When flies are chronically exposed to paraquat during adulthood (data not shown), they rapidly die, eliminating the ability to detect interactions between both herbicides. Paraquat has a much more potent effect on organisms, and therefore, we decided that chronic exposure to atrazine, and a short exposure to paraquat, would be ideal for our experiment.

### 2.2. Climbing Ability

After 24-hour paraquat (or control) exposure, the flies, in groups of 10, were transferred to new empty vials, and within one to two hours, were assayed for climbing ability with a Rapid Iterative Negative Geotaxis (RING) apparatus based on the approach in [31]. Our apparatus holds six vials vertically and allows for low-impact, concurrent contact with a table surface, after which the flies begin to climb. The measured intervals from 0.5 cm to 7 cm, in 0.5 cm increments, are visible through the vials and allow estimates of climbing height. Each block of climbing trials was performed in a randomized order at room temperature on the same benchtop, between 1500 and 1800 hours of a single day, in order to minimize environmental variation. The climbing trials were videotaped, and climbing height was scored three seconds after the last impact. The flies that did not show any movement for the duration of the video and flies that were not knocked to the bottom of the vials were excluded from data collection. Vials were assayed in a random order. The data were collected over two independent blocks. Combined across both blocks, the sample size for each sex within each treatment was approximately 180 flies.

The effects of treatment on climbing height were analyzed in R version 3.4.0 using linear models, TukeyHSD for post hoc analysis, and the Anova function from the *car* package for simultaneous model term effects [32,33]. The models included atrazine and paraquat concentrations as independent factors, along with their interaction. The factors vial and block were not included in the final models because they were not significant (*p* > 0.05). The sexes were analyzed separately. Data were analyzed in two groups, separated by paraquat concentrations (either 200 µM or 5 mM paraquat), and considered with relevant control and atrazine treatments. One group consisted of treatments: 1, 2, 4, and 5 (Table 1), while the other consisted of: 1, 3, 4, and 6.

The goal of the study was to examine what happens when organisms are exposed to both atrazine and paraquat, not to compare the independent effects of these substances. The statistical model used here allows for testing of the independent effects of each concentration of atrazine or paraquat. For example, a significant effect of paraquat in the low dose group indicates that, on average, flies exposed to 200 µM paraquat had different climbing ability to flies fed 1% sucrose. The only direct comparison of the effects of atrazine and paraquat is in the interaction term. The literature lacks consensus in relation to a definition of interaction [4,6]. In this study, interactions are referred to in comparison to an additive effects model, as described recently by [6] for factorial studies, and account for magnitude and response direction. If a significant statistical interaction between atrazine and paraquat is found, this indicates either synergy or antagonism. 

### 2.3. Longevity

After the 24-hour paraquat (or control) exposure, flies were transferred back to vials with the same type of food that they were reared on and any dead flies at this point were counted (day 1). Dead flies in each vial were counted every day at approximately the same time for 30 days. From day 31 until day 61, dead flies were counted every other day. Flies were transferred to fresh food every five to six days. The data were collected over three independent blocks. All blocks lasted at least 60 days. Blocks 1 and 2 lasted 61 days, and block 3 lasted 60 days. The flies were discarded after these times, making these data right-truncated. Combined across all blocks, the sample size for each group of flies (flies of one sex belonging to one of the six treatments) was about 250 to 300 flies.

The effects of treatment on longevity were analyzed in R version 3.4.0 using a Cox Regression with the coxph function from the *survival* package [33,34]. The models included atrazine and paraquat concentrations as independent factors along with their interaction. Block was included as a random factor in the model using the frailty function in the *survival* package. Vial was not included in the final models as it was not significant. The sexes were analyzed separately, and the blocks were analyzed together as well as separately (Tables S1–S4). The data were analyzed in two groups, separated by paraquat concentrations (either 200 µM or 5 mM paraquat), as was done with climbing data.

## 3. Results

### 3.1. Climbing Ability

**Figure 1:**
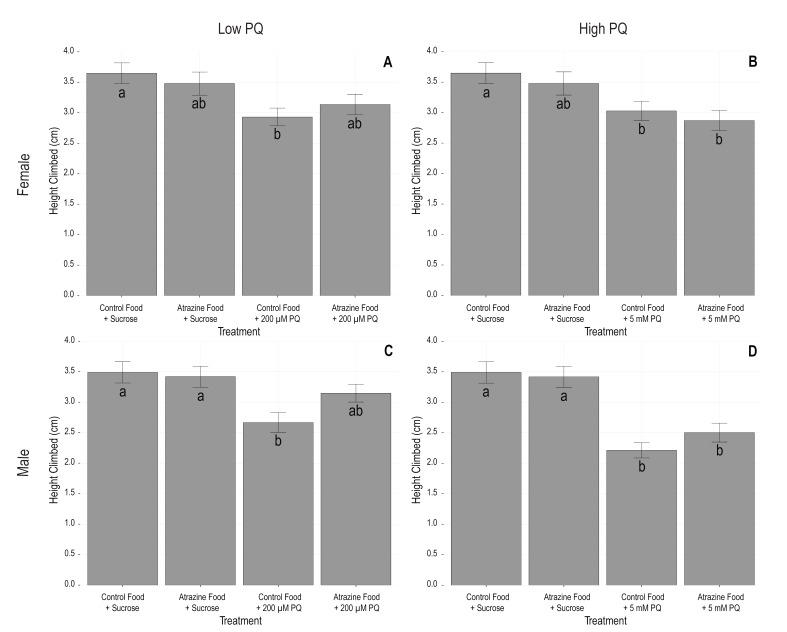
Mean climbing ability (cm) is shown 3 seconds after impact in the RING apparatus for (**A**) female flies in the low (200 µM) paraquat treatment, (**B**) female flies in the high (5 mM) paraquat treatment, (**C**) male flies in the low (200 µM) paraquat (PQ) treatment, and (**D**) male flies in the high (5 mM) paraquat treatment. Control and atrazine means are the same in panels **A** and **B** and in panels **C** and **D**. Bars indicate standard errors. Letters on each bar denote significant differences as determined by post hoc Tukey tests.

Paraquat exposure had a significant effect on climbing ability. Both the 200 µM and 5 mM concentrations of paraquat had consistently strong, negative effects on climbing height in both sexes (Figure 1, Table 2 and Table 3). Atrazine did not have an independent effect on climbing height in either sex (Figure 1, Table 2 and Table 3). Interestingly, the climbing height of flies reared on atrazine food and then exposed to 200 µM paraquat was not significantly different from controls (Tukey; Female *p* = 0.139, Male *p* = 0.442), whereas, the climbing height of flies exposed to 200 µM paraquat but reared on food without atrazine was significantly reduced compared to controls (Tukey; Female *p* = 0.010, Male *p* = 0.002, Tables S5 and S6). This suggests that there may be a weak mitigative effect of atrazine via a negative (−) antagonistic interaction with paraquat. There was not a significant atrazine by paraquat interaction (Female *p* = 0.267, Male *p* = 0.089), although it was close for males.

### 3.2. Longevity

**Figure 2:**
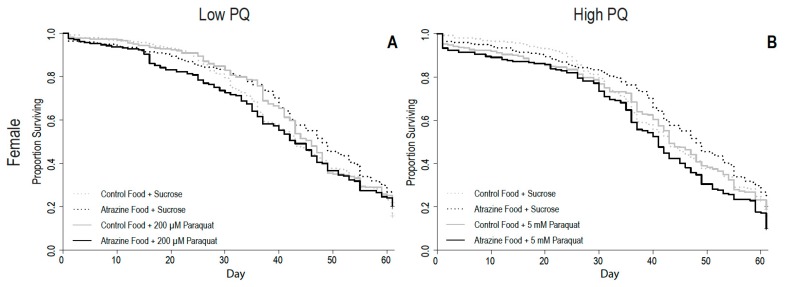
Survival as a proportion of original flies alive over time for (**A**) female flies in the low (200 µM) paraquat (PQ) treatment and (**B**) female flies in the high (5 mM) treatment. Control and atrazine survival lines in panels A and B are the same.

For female longevity, there was a significant interaction between atrazine exposure and each concentration of paraquat (atrazine and 200 µM paraquat *p* = 0.0056, atrazine and 5 mM paraquat *p* < 0.001, Table 4). This, combined with the hazard ratios, indicates a positive (+) synergistic interaction such that combined atrazine and paraquat exposure decreases longevity while the individual exposures either have no effect or a weak protective effect on longevity (Figure 2, Table 4). There was no significant three-way interaction (atrazine by paraquat by block; *p* = 0.794 for 200 µM paraquat; *p* = 0.358 for 5 mM paraquat) in females, indicating that the combined effect of atrazine and paraquat was generally consistent between blocks. Analyses for individual blocks are presented in Tables S1 and S2. In males, however, there was a significant three-way interaction (atrazine by paraquat by block) for longevity in both paraquat treatments (*p* < 0.001 for 200 µM paraquat; *p* = 0.015 for 5 mM paraquat). Because this means that the effect of the interaction is generally not consistent, we were not comfortable making a determination on its significance. Therefore, male survival curves and statistical analysis are presented in Figure S1 and Table S7. The individual analyses for each block are presented in Tables S3 and S4.

## 4. Discussion

Our results suggest that combined exposure to atrazine and paraquat can have significant effects on *D. melanogaster*; effects that are different than the sum of exposures to each individual herbicide. The combination of atrazine and paraquat exposure significantly decreased longevity in females, compared to the exposure of either herbicide alone, suggesting a synergistic effect between the two chemicals. In contrast, atrazine appears to have weak mitigative effect against the negative effects of paraquat on climbing ability. Our results show that interactions do occur between atrazine and paraquat in *D. melanogaster*, and that the effects can vary depending on the trait investigated. 

We are unaware of any studies that examine the combined effects of atrazine and paraquat, although there is evidence of interactions between atrazine or paraquat and other factors. For example, paraquat and the fungicide maneb affect stress response genes [35] and motor ability [27]. Heat stress [36] and diet [26] also interact with paraquat to affect survival. Several studies have found synergistic interactions between atrazine and organophosphate or pyrethroid insecticides on invertebrates [23,37,38]. Atrazine and cadmium interact to increase lipid peroxidation [24], which can cause cell damage. In our study, the interactive effect on longevity between paraquat and atrazine may have resulted from oxidative stress. In animals, paraquat is known to cause mitochondrial oxidative stress [17], which in flies, reduces longevity [18,19,20]. Atrazine has been shown to cause differential expression of proteins associated with oxidative stress [15], as well as increase oxidative damage, increase reactive oxygen species generation, and alter antioxidant gene expression in flies [13]. This suggests that, like paraquat, atrazine induces oxidative stress. Oxidative stress and mitochondrial function are important factors in longevity and aging [39,40,41,42,43]. Although oxidative stress was not directly measured in this study, the synergistic effect that was observed on longevity may be a result of the compounding oxidative stress that each chemical independently causes over an extended time period. 

In addition to affecting longevity in model systems, stressors such as herbicides have also been shown to affect motor ability [44,45,46,47,48]. In our study, we consistently find negative, independent effects of paraquat, but not atrazine, on motor ability. Motor ability is closely connected to the functioning of the dopaminergic system—death of dopaminergic neurons and disruption of the system cause motor deficiencies. There is evidence that atrazine and paraquat independently affect dopaminergic functioning. In rats, atrazine exposure has been shown to disrupt the dopaminergic system through changes in neuron count and neurotransmitter levels [9,44,47,48,49,50], which is likely to be the underlying reason for observed locomotor deficiencies in those studies [44,48]. In rats and flies, paraquat disrupts the dopaminergic system and causes locomotor deficiencies [19,22,51,52]. Therefore, we predicted that combined exposure to both herbicides may decrease climbing ability more severely than the individual exposures. Unexpectedly, this is not what we observed. In fact, our results suggest that atrazine may have a weak (*p* = 0.089) mitigative effect against paraquat’s influence on climbing ability. Pre-exposure to atrazine has been shown to cause the mosquito, *Aedes aegypti*, to be more tolerant to organophosphate insecticides, such as temephos [53,54], with some suggestion that atrazine-induced upregulation of cytochrome P450 genes might be responsible [53]. It appears that *D. melanogaster* could be primed by chronic atrazine exposure to respond more effectively to acute paraquat exposure, at least initially. Over a longer time period, it appears that the combination of atrazine and paraquat is detrimental to survival.

Our results also suggest that mating status may influence the effects of environmental stressors. In this study, exposure to atrazine increased survival in females (Table 4), while in a previous study from the laboratory, using the same atrazine concentration, exposure decreased female longevity [14]. One possible explanation for this difference is that the flies in this study were kept as virgins throughout the experiment, while in [14] they were allowed to mate freely. Female *D. melanogaster* that have mated show reduced longevity compared to those that have not [55]. It is thought that this occurs because of physical stress [56], chemical stress [55,57,58], and the increased energetic cost of producing eggs [58,59]. There is also evidence to suggest that reproduction may be a cause of oxidative stress [42], which is an important factor in longevity and aging. Reproduction has many effects on males and females that affect their longevity, and may explain the difference between our observed effects of atrazine and those of [14].

Although studies examining the effects of stressor interactions have increased in recent years, there are many combinations of stressors that have yet to be examined at all. The usage areas of many pesticides overlap, meaning that organisms could be exposed to multiple chemicals during their lifetime. It is likely that organisms experience many additional, unknown interactions between biotic and/or abiotic stressors that have not been characterized. Furthermore, most studies have either investigated only a single genotype or have not controlled for genetics, and thus we do not know whether there is genetic variation segregating in natural populations for responses to multiple stressors. A better understanding of these possible interactions will allow us to predict how populations may respond to complex anthropogenic changes.

## 5. Conclusions

Exposure to multiple stressors can affect organisms in ways that are different than the sum of their individual effects and these interactions are often difficult to predict. The herbicides atrazine and paraquat interact and affect climbing ability and longevity in different manners when investigated in *D. melanogaster*. Atrazine appears to have a weak mitigative effect against paraquat’s negative influence on climbing ability. Atrazine and paraquat have a synergistic effect on longevity. This system can serve as a model to help us better understand the consequences of stressor mixtures and how anthropomorphic changes can affect traits related to fitness in other organisms.

## Figures and Tables

**Table 1:** Treatment groupings that were tested for climbing ability and longevity.

Treatment	Food	Adult Exposure ^†^
1	Control Food	1% sucrose
2	Control Food	200 µM paraquat in 1% sucrose
3	Control Food	5 mM paraquat in 1% sucrose
4	2 ppM Atrazine Food	1% sucrose
5	2 ppM Atrazine Food	200 µM paraquat in 1% sucrose
6	2 ppM Atrazine Food	5 mM paraquat in 1% sucrose

^†^ Sucrose or paraquat fed on gel blot paper.

**Table 2:** ANOVA table for female climbing height in low (200 µM) and high (5 mM) paraquat treatments.

Grouping	Factor ^†^	d.f.	Sum of Squares	F-Value	*p*-Value ^§^
200 µM PQ	Atz	1	0.05	0.012	0.912
	200 µM PQ	1	45.47	10.5098	**0.0013**
	Atz: 200 µM PQ	1	5.34	1.2344	0.267
	Residuals	609	2634.93	-	-
5 mM PQ	Atz	1	3.99	0.924	0.337
	5 mM PQ	1	56.38	13.046	**<0.001**
	Atz: 5 mM PQ	1	0.01	0.0014	0.970
	Residuals	593	2562.78	-	-

^†^ Atz indicates atrazine, PQ indicates paraquat, d.f. indicates degrees of freedom. ^§^ Bold indicates statistical significance.

**Table 3:** ANOVA table for male climbing height in low (200 µM) and high (5 mM) paraquat treatments.

Grouping	Factor ^†^	d.f.	Sum of Squares	F-Value	*p*-Value ^§^
200 µM PQ	Atz	1	7.44	1.753	0.186
	200 µM PQ	1	46.90	11.055	**<0.001**
	Atz: 200 µM PQ	1	12.30	2.898	0.089
	Residuals	637	2702.34	-	-
5 mM PQ	Atz	1	2.2	0.560	0.455
	5 mM PQ	1	193.3	49.270	**<0.001**
	Atz: 5 mM PQ	1	5.3	1.351	0.246
	Residuals	639	2507.0	-	-

^†^ Atz indicates atrazine, PQ indicates paraquat, d.f. indicates degrees of freedom. ^§^ Bold indicates statistical significance.

**Table 4:** Cox regression analysis for female longevity in low (200 µM) and high (5 mM) paraquat treatments.

Grouping	Term ^†^	Hazard Ratio ^‡^	*p*-Value ^§^
200 µM PQ	Atz	0.685	**<0.001**
	200 µM PQ	0.838	0.071
	Atz: 200 µM PQ	1.507	**0.0056**
	Block	-	**<0.001**
5 mM PQ	Atz	0.709	**0.0013**
	5 mM PQ	0.839	0.067
	Atz: 5 mM PQ	1.822	**<0.001**
	Block	-	**<0.001**

^†^ Atz indicates atrazine, PQ indicates paraquat. ^‡^ Hazard ratios below 1 indicate the term enhances survival while hazard ratios greater than one indicate the term decreases survival. ^§^ Bold indicates statistical significance.

## Supplementary Materials

The following are available online at https://www.mdpi.com/2075-4450/10/11/398/s1, Table S1: Separate block cox regression for female longevity-low PQ, Table S2: Separate block cox regression for female longevity-high PQ, Table S3: Separate block cox regression for male longevity-low PQ, Table S4: Separate block cox regression for male longevity-high PQ, Table S5: Post hoc Tukey comparisons for female climbing-low PQ, Table S6: Post hoc Tukey comparisons for male climbing-low PQ, Table S7: Cox regression analysis for male longevity, Figure S1: Male survival curves, Data S1: Raw climbing data, Data S2: Raw longevity data.
